# Deterioration Mechanisms and Advanced Inspection Technologies of Aluminum Windows

**DOI:** 10.3390/ma15010354

**Published:** 2022-01-04

**Authors:** Huaguo Chen, Cheuk Lun Chow, Denvid Lau

**Affiliations:** Department of Architecture and Civil Engineering, City University of Hong Kong, Hong Kong, China; c.huaguo@my.cityu.edu.hk (H.C.); cheuchow@cityu.edu.hk (C.L.C.)

**Keywords:** aluminum windows, artificial intelligence, deterioration mechanisms, environmental factors, molecular dynamics simulation, inspection techniques

## Abstract

Aluminum windows are crucial components of building envelopes since they connect the indoor space to the external environment. Various external causes degrade or harm the functioning of aluminum windows. In this regard, inspecting the performance of aluminum windows is a necessary task to keep buildings healthy. This review illustrates the deterioration mechanisms of aluminum windows under various environmental conditions with an intention to provide comprehensive information for developing damage protection and inspection technologies. The illustrations reveal that moisture and chloride ions have the most detrimental effect on deteriorating aluminum windows in the long run, while mechanical loads can damage aluminum windows in a sudden manner. In addition, multiple advanced inspection techniques potential to benefit assessing aluminum window health state are discussed in order to help tackle the efficiency problem of traditional visual inspection. The comparison among those techniques demonstrates that infrared thermography can help acquire a preliminary defect profile of inspected windows, whereas ultrasonic phased arrays technology demonstrates a high level of competency in analyzing comprehensive defect information. This review also discusses the challenges in the scarcity of nanoscale corrosion information for insightful understandings of aluminum window corrosion and reliable window inspection tools for lifespan prediction. In this regard, molecular dynamics simulation and artificial intelligence technology are recommended as promising tools for better revealing the deterioration mechanisms and advancing inspection techniques, respectively, for future directions. It is envisioned that this paper will help upgrade the aluminum window inspection scheme and contribute to driving the construction of intelligent and safe cities.

## 1. Introduction

Aluminum windows are essential parts of building envelopes, as they establish a connection/separation between indoor rooms and the external environment [1,2]. Due to their importance, aluminum windows should satisfy technical requirements for practice. The requirements of a building fenestration system are generally sound air permeability, water tightness, and wind-load resistance. The air permeability performance of aluminum windows has a great impact on indoor heat loss and energy efficiency [3]. The watertight performance of aluminum windows reflects their effectiveness in preventing the intrusion of rainwater into internal buildings [4]. Wind-load resistance refers to the ability of windows to withstand wind pressure without any mechanics-induced damage (e.g., cracking, local yield, and bond failure) or functional impairment such as loose hardware and opening difficulties [5,6]. These performance indicators provide valuable information for ensuring the quality of aluminum windows at the product stage. When aluminum windows are installed in buildings, long-term exposure to complicated environments can affect their quality and working performance.

Aluminum windows continuously suffer from various mechanical and environmental influences in service [7,8,9]. Mechanical loadings such as strong wind, seismic loads, and impact force cause deformation and failure of aluminum windows in a sudden manner. In addition to mechanical damage, aggressive environmental factors can gradually deteriorate an aluminum window. Among these environmental factors, chloride ions, moistures, and the combination of both are the leading causes of aluminum window deterioration, especially in offshore and marine areas [10,11]. Although the aluminum surface is covered by thin aluminum oxide film, chloride ions and moisture can still penetrate this film in the long run [10,12]. These aggressive factors participate in the electrochemical corrosion of aluminum. Eventually, different kinds of corrosion types form in aluminum windows, predominantly with pitting [13]. Among the constitutes of aluminum windows, connections such as hinges and handwaves are the most sensitive components to corrosion because of their large specific surface area. In addition to the effect of external environments, their intrinsic material properties also influence the degradation of aluminum windows. For example, glass is a brittle material that becomes fragile when suffering from loading. Aluminum alloy, the primary material for aluminum window frames, is sensitive to corrosion when exposed to corrosive environments. It is seen clearly that both external environmental conditions and material intrinsic properties can influence the degradation of aluminum windows in different manners. A comprehensive understanding of different degradation mechanisms can offer first-hand information that will help when inspecting aluminum windows for the most fragile parts.

Aluminum window inspection is a routine yet necessary maintenance task that ensures a building’s safety, functionality, and aesthetics, since degradation and defects in these windows can reduce the service life of buildings. To diagnose the health condition of aluminum windows, many countries or regions require a periodical and standardized inspection. Traditional visual identification of defects in aluminum windows requires a qualified person to complete the process with simple equipment such as a telescopic inspection mirror. This conventional process is inefficient, time-consuming, and labor-intensive. Even worse, some aluminum windows that have been inspected have still fallen onto the street, because invisible defects growing on inaccessible surfaces cannot be detected by direct visual inspection. Due to the negligence of window defects, malfunctioning windows can lead to life-threatening events if they fall from buildings and hit pedestrians. To prevent this from happening, many non-destructive testing (NDT) technologies have great potential for promoting the accuracy and efficiency of aluminum window defects inspection. NDT technologies are strategic procedures for gathering information on structure conditions and existing damage and defining adequate remedial measures; they have a powerful ability to assess health state in a non-invasive manner [14,15,16]. These advanced NDT technologies include ultrasonic thickness measurement technology, ultrasonic guided wave inspection, and active infrared thermographic inspection. Ultrasonic testing measurements can be used to inspect the metal thickness and locate the defect dimensions [17,18,19]. Infrared thermal images can be employed to detect the defect profiles in inspected objects [20,21,22]. Although these mature technologies have been applied in many fields, the feasibility of their application in aluminum window inspection is reliant on understandings of their usage and functions.

In view of the lack of a comprehensive survey concerning different degradation mechanisms, this paper aims to illustrate the degradation mechanisms of aluminum windows subjected to different external conditions, including mechanical loadings and environmental factors. The focus will be the effect of humidity and chloride ions, since these are the most influential factors in the deterioration of aluminum windows. In addition, intelligent inspection technologies will be introduced in order to address the efficiency problem with direct visual inspection. This review also discusses the challenges in the scarcity of molecular corrosion information for insightful comprehension of aluminum window corrosion and trustworthy window inspection tools for lifespan prediction. In this regard, the in-depth understanding of the degradation mechanisms of aluminum windows can be comprehensively enriched through MD simulations in the future. Meanwhile, the utilization of artificial intelligence technologies will promote high accuracy and efficiency in inspection and prediction tasks for aluminum windows. It is envisioned that a comprehensive overview of the existing understandings and future perspectives of aluminum windows will help ascertain the fragile parts in these windows and upgrade the aluminum window inspection scheme as we move toward intelligent cities.

## 2. Deterioration of Aluminum Windows

Aluminum windows work in a complicated environment with multiple aggressive factors such as chloride ions, humidity, temperature, and mechanical loadings [23]. Different factors deteriorate aluminum windows and affect their working performance in different ways. This section highlights current understandings of the deterioration mechanisms of aluminum in various environments. This knowledge is expected to help prevent window deterioration in a specific environment in an intrinsic manner.

### 2.1. Moisture

In the process of aluminum window corrosion, moisture is one of the most influential factors. When water comes into contact with an aluminum substrate, a protective barrier, namely aluminum oxide, between the aluminum and the surrounding medium forms almost instantly [24]. After the formation of oxide film, water molecules from a humid environment can be deposited at the surface of the aluminum oxide via condensation and absorption [25]. Water condensation on the surface of aluminum windows, followed by the formation of moisture film, is an essential prerequisite to the corrosion of aluminum windows. Water films with a thickness of less than a few hundred micrometers act as containers and transport for corrosive species. It has been found that a critical relative humidity threshold in the range of 40–70% leads to the formation of an electrolyte film on the surface of aluminum. When the critical value is not in this range, practically no corrosion occurs at room temperature. When water penetrates into aluminum oxide film, the reaction between aluminum and water can cause decohesion of the thin aluminum oxide film and the formation of a bubble in which the vapor pressure is large. Furthermore, repeated penetration of the moisture coating allows oxygen to reach the metal surface, boosting cathodic reactions and, as a result, causing the anodic dissolution of aluminum ions. The penetration of water molecules induces electrochemical reactions under the aluminum oxide film.

**Figure 1:**
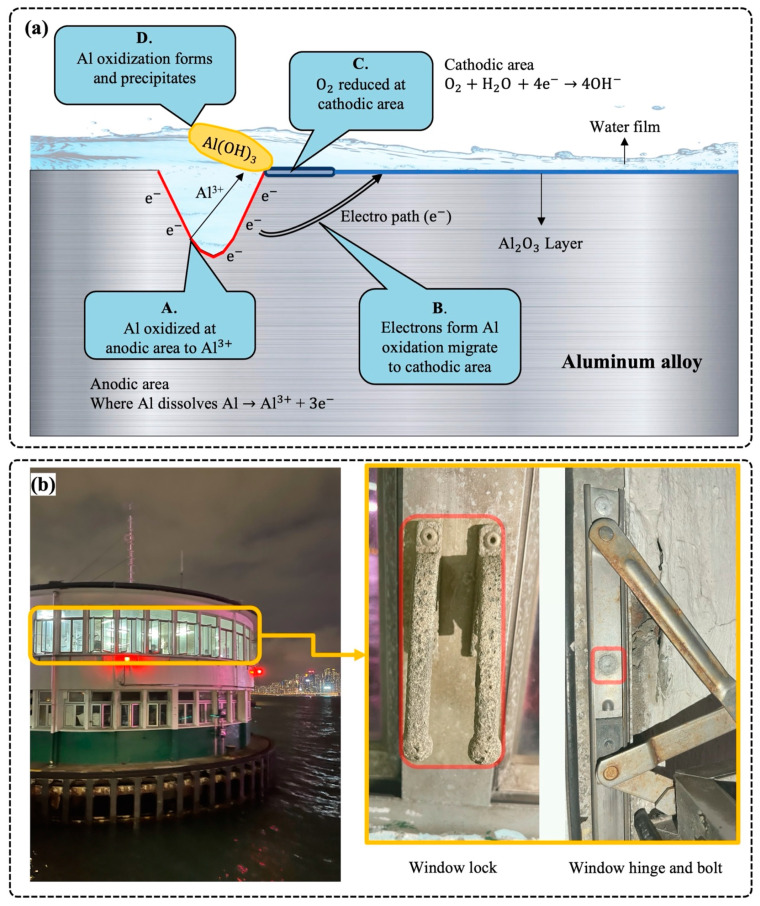
The effect of moisture and chloride ions on aluminum window. (**a**) Schematic illustrations for electrochemical reactions at the aluminum interface. (**b**) The connection components, namely the lock, hinge, and bolt of the aluminum window, corrode in salt environments.

Figure 1a illustrates the typical process of aluminum corrosion. Such electrochemical corrosion starts with electrical charge transfers and ends with the formation of aluminum oxidization. First, aluminum is oxidized to aluminum ions in anodic areas, with the following chemical equation.

(1) $$\mathrm{Al}\;-3e^{-}\;\to \;{\mathrm{Al}}^{3+}$$

Secondly, the electrons released from the oxidation reaction migrate to cathodic areas. In this area, oxygen is an oxidant that promotes a cathodic reaction. The corrosion rate is strongly influenced by the oxygen content of the water. The oxidation reaction is dependent upon the solution’s pH value. When the pH of the surrounding solution is neutral or alkaline, the reaction is shown as follows.

(2) $$O_{2}+2H_{2}O+4e^{-}\;\to \;4{\mathrm{OH}}^{-}\;$$

In acid solution, meanwhile, the reaction is as follows.

(3) $$O_{2}+4H^{+}+4e^{-}\;\to \;2H_{2}O\;$$

Here, we take the reaction in neutral or alkaline solution as an example. Following this, the oxygen in the cathodic area gains the electrons, followed by a reduction reaction. Eventually, different kinds of corrosion formations, such as pitting corrosion and exfoliation corrosion, lead to the degradation of aluminum alloy materials. The above mechanisms of aluminum corrosion under a moisture environment demonstrate that the existence of oxide film at aluminum alloy surface is the main reason for the atmospheric corrosion resistance of aluminum alloy, and the corrosion resistance of aluminum is determined by the physical-chemical stability of the oxide coating.

### 2.2. Chloride Ions

In addition to humidity, aluminum windows are highly susceptible to corrosion attacks in chloride-containing media [26]. Salt contaminants with high humidity can trigger corrosion in atmospheric environments when aluminum windows work in offshore, marine, or salt-rich regions [27,28]. Initially, moisture-carrying salt contents penetrate the protective aluminum layers of the window surface. The incorporation of chloride ions directly facilitates the aluminum corrosion process by altering the corrosion potential [29]. Since this unique solution functions as an electrolyte and there are potential chemical differences between aluminum and alloyed metal, electrochemical reactions are formed. A high chloride ion concentration leads to a low negative corrosion potential and low general corrosion resistance [23]. The type of cation combined with chloride ion also plays a vital role in facilitating the corrosivity of chloride ions, with an order of CaCl_2_ > MgCl_2_ > NaCl [30], as the hydrolysis of chloride salts leads to a decreased pH value in localized areas. The acidic and alkaline solutions are responsible for the instability of the aluminum oxide layer [26]. Aluminum ions dissolve in the microstructure as the increasing penetration depth of the chloride ions presents in the solution. With the development of the porous microstructure of the aluminum material, the corrosion resistance of the windows decreases [31]. Eventually, some parts of the aluminum windows, such as the locks, hinges, and bolts, corrode in the form of pitting corrosion, which is the most common corrosion [32], as presented in Figure 1b. Corrosion products facilitate the penetration of moisture and chloride ions by capillarity and retention due to surface tension phenomena. Despite the significant quantity of studies on the effect of chloride ions on corrosion potential change and microstructure, the basic mechanisms of chloride ion movements and interaction at the nanoscale have not yet been ascertained. One possible theory is that chloride ions are the most mobile among the factors engaged in these interactions, owing to their tiny size [33]. Specifically, chloride ions substitute for oxygen atoms in the network of aluminum oxide films. As a result of this replacement, the film resistivity decreases, and the rate at which aluminum atoms diffuse into the solution increases. Another theory involves chloride ion adsorption on the oxide surface, chloride ion penetration through the oxide coating, and localized aluminum dissolution at the metal/oxide interface [34]. Both of these hypotheses are founded on assumptions and lack solid evidence to back them up.

### 2.3. Air Pollutants

Fuel combustion mainly generates air pollutants such as ${\mathrm{SO}}_{2}$, ${\mathrm{NO}}_{2}$, and NO in the atmosphere. ${\mathrm{SO}}_{2}$, ${\mathrm{NO}}_{2}$, and NO are not very reactive as gases at room temperature, except in the presence of moisture. When the relative humidity is high, they can react with water molecules in the atmosphere and attack aluminum alloy materials [35]. The reactions between water and air pollutants produce sulfate or nitrate ions. The presence of these ions changes the pH value of the solution and can locally destroy aluminum films, which are unstable at pH < 4 and pH > 9 [36], as shown in the following chemical equation:

(4) $${\mathrm{Al}}_{2}O_{3}+H_{2}{\mathrm{SO}}_{4}\;\to \;{\mathrm{Al}}_{2}{\left({\mathrm{SO}}_{4}\right)}_{3}+H_{2}O$$

(5) $${\mathrm{Al}}_{2}O_{3}+{\mathrm{HNO}}_{3}\;\to \;\mathrm{Al}{\left({\mathrm{NO}}_{3}\right)}_{3}+H_{2}O$$

The aluminum ions dissolving from aluminum films are absorbed and react with sulfate or nitrate ions. These reactions finally produce stable and insoluble aluminum sulfate compounds or aluminum nitrate compounds, as shown in the following chemical equation:

(6) $$x{\mathrm{Al}}^{3+}+\;y{\mathrm{SO}}_{4}^{2-}+\;z{\mathrm{OH}}^{-}\to {\mathrm{Al}}_{x}{\left({\mathrm{SO}}_{4}\right)}_{y}{\left(\mathrm{OH}\right)}_{z}$$

(7) $$x{\mathrm{Al}}^{3+}+\;y{\mathrm{NO}}_{3}^{-}+\;z{\mathrm{OH}}^{-}\to \;{\mathrm{Al}}_{x}{\left({\mathrm{NO}}_{3}\right)}_{y}{\left(\mathrm{OH}\right)}_{z}$$

These produced compounds acting as filler can occupy the previously corroded positions and contribute to the strong anticorrosion ability of aluminum and aluminum alloys after the air pollutants are oxidized [37]. It was found that Al dissolution was affected by the molecular interaction between anions and aluminum instead of the solution pH [38]. In addition, the ${\mathrm{SO}}_{2}$, ${\mathrm{NO}}_{2}$, and NO gases have a synergistic effect on the corrosion of aluminum and its alloys in the atmosphere [39]. The existence of ${\mathrm{SO}}_{2}$’s reaction products, sulfate ions, make it substantially less aggressive than chlorides, as their reaction products with water are highly solvated ions with a large diameter. The atomic radius of sulfate ions is 149 nm, eight times larger than that of chlorides. The large volume of sulfate ions impedes their penetration into aluminum. As for nitrate ions, despite the fact that they have the same rate of diffusion as chlorides in oxide film, they also have the ability to repair the corroded aluminum as a result of their oxidizing abilities. The above analysis accounts for the fact that the effect of ${\mathrm{SO}}_{2}$, ${\mathrm{NO}}_{2}$, and NO in aluminum corrosion is relatively small compared to that of moisture and chlorides [36].

### 2.4. Temperature

Similar to most common metals, aluminum and its alloy are less vulnerable to temperature than to pollution. The temperature has no significant impact on the atmospheric corrosion of aluminum and its alloys at a normal atmospheric temperature of 20–40 °C, according to the results of previous studies [40]. Beyond the above temperature range, the corrosion products pertaining to aluminum oxides and the chemical potential of aluminum are temperature-dependent. Specifically, at a temperature over 40 °C, the aluminum oxide film reacting with the aqueous solution tends to thicken and experience polymorphic changes: formation of monoclinic aluminum trihydroxide (60–90 °C), orthorhombic aluminum oxyhydroxide (above 90 °C), and hexagonal aluminum oxide (above 350 °C) [33]. These oxidated products alter the stability of aluminum oxide film in a different way, but the in-depth understanding of their role in the corrosion process is still unknown. As for the chemical potential, the pitting potential is one of the most important features of aluminum alloy. Pitting potential drops linearly with temperature, with a transition value at 30 °C, beyond which there is a substantially steeper reduction [41]. This can be linked to the different products in passive film structure with temperature, alteration, and pitting potential [42].

**Figure 2:**
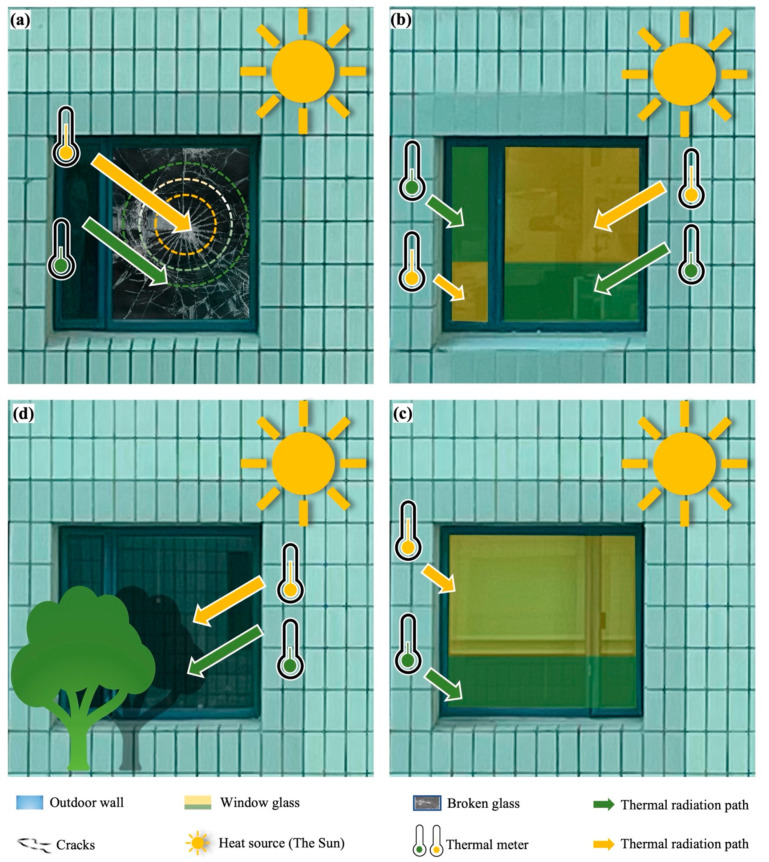
The effect of temperature on window glass. (**a**) Thermal gradient leading to crack initiation and growth of window glass. (**b**–**d**) The common causes of glass crack initiation and growth are glass color, indoor shading devices, and outdoor shading patterns.

Compared with aluminum alloy, glass is more sensitive to environmental temperature than aluminum materials due to its poor thermal conductivity [43]. When glass systems are exposed to sunlight and other heat sources, one area of a glass pane becomes hotter than its adjacent areas. The temperature variation in a glass pane leads to thermal stress and increases the risk of glass breakage, as displayed in Figure 2a [44]. When the thermal stress exceeds the critical breaking stress threshold, glass breakage occurs [45,46]. The common causes of glass crack initiation and growth are glass color, outdoor shading patterns, and indoor shading devices, as depicted in Figure 2b–d [47]. Specifically, glass with tinted colors absorbs solar radiation [48]. Due to this absorption, colored glass is more likely to shatter owing to temperature stress than clear glass. The outdoor shading pattern is one of the most dynamic aspects, because shade patterns fluctuate annually. One practical solution to address this issue is to reduce the number of places where the glass panel is shaded by less than 50%. This will assist in preventing excessive temperature gradients. Indoor shading devices, such as blinds or drapes, can raise the temperature of the glass by a few degrees. This happens when a blind or shade reflects sunlight back through the glass, limiting heat convection and conduction away from the glass.

### 2.5. Mechanical Loadings

In addition to environmental factors, mechanical loads can have a destructive effect on structure components [49,50]. Wind and seismic loads are the key mechanical loads in aluminum window frameworks and glass, and glass is relatively fragile and prone to fracture [51,52]. Wind can cause damage to window glass in high-rise buildings as wind pressure increases with height. The breakage of window glass and its consequent interior damage due to wind and water penetration have been observed on several occasions. Wind affects aluminum windows in the form of out-of-plane loading. Although the wind load is a mechanical load, a particular stress level cannot solely induce glass failure. Instead, it is damage accumulation (static fatigue) mechanisms that dominate glass breakage [43]. The damage accumulation rate is heavily reliant upon the skewness and amplitude of probabilistic profiles. In other words, glass failure is a time-dependent reduction of material properties due to the integration of loading magnitude and duration. An effective prevention approach for window glass breakage is the use of window glass film. The safe film has a great capacity for repelling attempts at breakage under loading and even for blast forces. Compared to the glass plate, the aluminum framework has strong resistance to wind load, as the framework has high material properties such as tensile strength against wind and is normally fixed into the wall of the building [6,53]. Hence, strong connections between window frames and exterior building walls are important [54].

Under seismic loading, the aluminum window frames are subjected to both simultaneous in-plane and out-of-plane actions, which damages the windows in a serious manner. Specifically, earthquake-induced damage to window systems can cause casualty loss and result in considerable economic collapse. As an example, a building shakes from side to side when there is an earthquake, leading to the deformation of window frames. During deformation, the compression from the glass mounting frame causes crack initiation in window glass panes. The worst-case scenario is that the glass breaks and falls out of the window frames [55]. As a result of the partial or complete loss of window functionality, the thermal efficiency of buildings is impaired, and life is threatened. Previous occurrences show that damage to glazing systems is typically caused by a mismatch between the structural framing’s deformation characteristics and the cladding’s movement capacity, such as a lack of slip-accommodating links and inadequate perimeter connection width [56]. Two conceptual approaches can be used effectively to enhance the seismic performance of conventional glazing systems, namely, the impedance of imposed seismic motions by improper joint assembly and the enhancement of drift accommodation by the use of rounded corners [57].

### 2.6. Challenge of Current Studies in Aluminum Alloy Corrosion

While macroscopic investigations of aluminum alloys have been conducted to assess the degradation of aluminum windows, there are still limitations in understanding their degradation mechanisms under sole or joint environmental factors at a top-down scale. The degradation mechanisms under the effect of different factors have not yet been fully understood, as current understandings of resistance to aggressive species rely largely on macroscopic experiments such as condensing humidity tests (ISO 6270-1), salt spray tests (ISO 9227), and, Kesternich (ISO 3231, 0.2 L ${\mathrm{SO}}_{2}$) [58]. These traditional approaches provide anticorrosion performance indicators for evaluating corrosion resistance ability. Such assessment information is valuable for guaranteeing aluminum window quality in practice. However, conventional detection tools have difficulties in revealing the information behind the corrosion performance, as corrosion mechanisms generally stem from the molecular interaction between corrosive media and aluminum substrate at the nanoscale. The understanding of the molecular origin can help manipulate a sound material system for manufacturing strong and durable aluminum windows and prevent corrosion occurrence from the root when accurate methods are used. For example, the chloride inhibitors and the smart nanocontainers have already utilized the corrosion inhibition knowledge from the nanoscale and achieved impressive performance [59,60].

Apart from the lack of nanoscale corrosion information, precise and fast mimicking of corrosive environments is another key challenge. Traditionally, corrosion coupon testing and climatic chamber testing are used to investigate general corrosion [61,62]. The former one in the form of measuring the mass loss of specimens requires long exposure periods of several years, while the latter considers only a few environmental factors. Specifically, in corrosion coupon testing, short exposure periods can yield unrepresentative corrosion rates, especially for aluminum alloys that form passive films. Climatic chamber testing tends to simplify a complex environment into one or two environmental variables. Environmental chambers with only a few environmental variables, such as moisture and chloride ions, are generally used in order to accelerate the corrosion of samples. As a result, a real corrosive environment cannot be reproduced [63].

## 3. Technologies for Aluminum Window Inspection

Since the traditional visual window inspection scheme presents difficulties with accurately detecting the health state of windows exposed to aggressive environments, the effort to replace traditional visual inspection with advanced non-destructive technologies should be paid more attention. This is especially the case for hidden subsurface and non-uniform corrosion which increases the risk of structural failure. Among the non-destructive technologies for defect testing, ultrasonic testing and infrared thermal image are the two predominant methods that provide an accurate picture of material defects. This section demonstrates their basic working principles and compares their respective advantages and disadvantages in order to help select an applicable technique for window inspection.

### 3.1. Ultrasonic Testing

**Figure 3:**
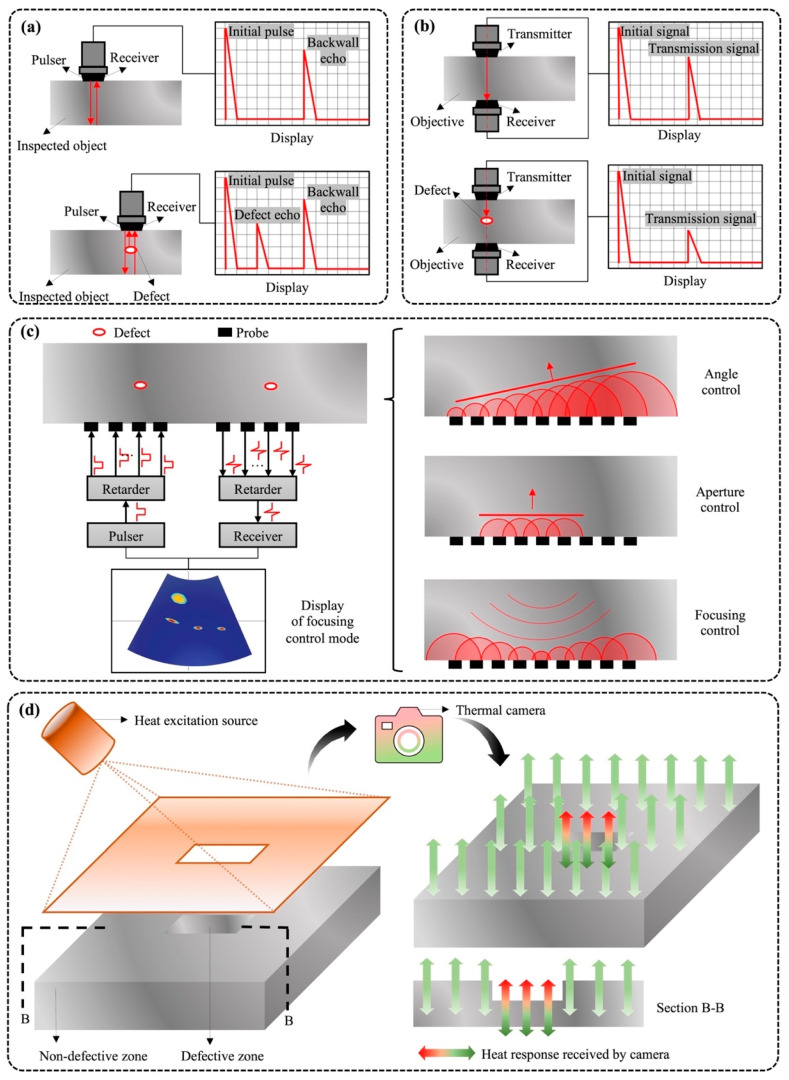
Advanced non-destructive technologies for aluminum window detections. (**a**) Ultrasonic wave transmission technology. (**b**) Ultrasonic wave reflection technology. (**c**) Phased array technology and phased array focal laws. (**d**) Active infrared thermal image.

Ultrasonic testing (UT) is a non-destructive detecting method (NDE) technique, based on the employment of short, high-frequency ultrasonic waves with a frequency above 20,000 Hertz [64,65]. This powerful technique has been used to detect and evaluate material defects at the surface, subsurface, and internal levels [66,67,68]. UT techniques can be categorized into conventional and advanced ultrasonic testing, in a historical sense. Conventional ultrasonic testing can be carried out in two ways: pulse-echo and through-transmission. Figure 3a shows the basic working principle of the ultrasonic pulse-echo testing technique. Initially, one piezoelectric transducer (transmitter) converts electrical energy into acoustic energy in the form of an ultrasonic wave [69,70]. The ultrasonic wave propagates in a detected object and reflects the signals when it reaches a discontinuity or boundary. The reflected signal travels back through the object to another transducer (receiver). The transmitter and receiver correspond to the pulse and echo, respectively. After the signal travels, the acoustic wave is converted to an electrical signal, which is then amplified. Based on this working principle, the defect echo signal is amplified and displayed on the screen when the defect is detected. In the display, the horizontal axis is proportioned to the travel time, and the vertical axis refers to the signal amplitude. The defect or material depth can be obtained by multiplying half of the wave round-trip time by the material sound velocity. Due to the simplicity of detecting the presence and location of defects, ultrasonic pulse-echo testing techniques have been widely used for internal defects detection during aluminum corrosion damage assessments [71,72,73]. In contrast to the arrangement of transducers in the ultrasonic pulse-echo testing technique, a pair of aligned transducers, transmitter and receiver, are located on opposite sides of the detected object in the through-transmission method. Figure 3b demonstrates the basic measurement principle of ultrasonic through transmission [74]. The transmitter sends an ultrasound signal, which is then detected by the receiver. Both transducers move in a synchronous manner during the detection process in order to scan the measured material structure line by line [75]. When a defect is encountered, the acoustic signal will be absorbed, reflected, or scattered, and a significantly attenuated or even no signal will be shown on a display monitor. Despite the ability to inspect the defects, utilizing these two traditional approaches requires a lengthy inspection time in order to attain an accurate and full picture of the defect profiles [76,77].

In contrast with traditional ultrasonic techniques, which use a single piezoelectric crystal to transmit or receive ultrasonic signals, advanced UT technologies equipped with multiple probes can be employed to address the above-mentioned problem of low efficiency. This ability may be attributable to the multiple probes being arranged in different arrays and geometries and enabling feasibility and adaptability in the inspection [78,79]. An example of these advanced UT techniques is the ultrasonic phased arrays technique; its working mechanisms are illustrated in Figure 3c. These piezoelectric probes are stimulated by properly time-delayed pulses in different forms in order to ensure that ultrasonic waves can propagate in the predefined area. Specifically, by tuning the transmission time of array elements, the direction and depth of wave propagation alter correspondingly. The adjustment of wave transmission leads to improvements in the feasibility and applicability of imaging defects located in regions difficult to access [80]. The typical arrangements of probes incorporate angle, aperture, and focusing control.

### 3.2. Active Infrared Thermal Image

In addition to the contact measurements of ultrasonic testing techniques, non-contact techniques are deployed to detect equipment flaws and material defects [81]. A typical example of non-contact techniques is active infrared thermography (or thermal image), which is considered the most practiced and employed condition monitoring tool for non-destructive inspection of conductive materials’ surface and sub-surface flaws [21]. Active infrared thermography is equipped with a heat excitation source to stimulate the thermal evolution when the detected object experiences temperature variation, as reflected in Figure 3d. The presence of an external heat source alters temperature distribution at the surface of the object. This important equipment enables active infrared thermography to exhibit another function, namely, quantitatively characterizing the physical and thermal properties of defects [81].

Active infrared thermography works on the principle of the physical phenomenon that any objective with a temperature over absolute zero emits infrared radiation [82,83]. The wavelength of such infrared radiation is in the range of 700 nm–1 mm, which is invisible. In order to make the infrared radiation visible, a thermal camera translates wavelengths from the infrared spectrum into a visual image. When an external heat source is injected into an object, the temperature distribution is dependent on the existence of a defect and its thermal properties. The thermal camera records both the temporal and spatial evolution of surface temperature from the moment of stimulation till stabilizing to ambient state. Specifically, the three possible cases are analyzed as follows. (a) If an object has no defects, the temperature field distribution is uniform, as the heat flow can diffuse evenly to the inside or at the surface. (b) If there are thermal insulation defects inside the object, the heat flow will be blocked at the defects, leading to heat accumulation and local hot areas with high temperatures. (c) If there are thermal conductivity defects inside the object, a local cold zone will appear on the surface of the object. The local temperature difference of the object will inevitably lead to different infrared radiation intensity. These differences in turn help with the identification of defects. Due to its powerful functions, infrared (IR) thermography has been applied to evaluate mechanical defects and elastic deformations in glass sheets, as well as corrosion defect characterization [84,85,86].

Transferring the obtained thermographic images to defect information is an important task for defect detection and handling. Among the existing thermographic processing approaches for defect characterization, thermal contrast-based techniques are the most widely used due to their ability to improve subsurface defect visibility. In addition, it can be used to extract corrosion information, i.e., defect sizing and location and thermal properties, i.e., diffusivity [87]. Thermal contrast-based techniques are based on the classical corrosion model. In this model, defects are linked to surface temperature evolution. However, this approach is applicable to finding an early time of defect appearance in only one-dimensional detected objective without affecting by 3D thermal diffusion complexity. In order to enhance defect detectability, multiple techniques have been developed recently, such as matrix factorization techniques, statistical techniques, phase-sensitive techniques, and artificial intelligence-based techniques [81].

### 3.3. Comparison

Table 1 lists the pros and cons of the above-mentioned methods in order to find a suitable inspection technique that can accurately provide information about the health state of aluminum windows. The evaluations of these techniques depend on the cost and time of the inspection, the experience of personnel, and the ability to detect defect depth when used for aluminum window inspection. Regarding inspection time and cost, ultrasonic wave transmission technology is cheaper than ultrasonic phased arrays or infrared thermography, as the latter two approaches require expensive equipment and highly experienced staff to calibrate the instruction parameters. In addition, traditional ultrasonic inspections are conducted manually. With the Internet of Things and computation power development, ultrasonic phased arrays and infrared thermography can be automatically controlled by predefined programs [88,89]. In terms of inspection efficiency, infrared thermography can rapidly give preliminary results about the inspected object, since it is a non-contact technique that enables the drawing of a whole scale picture, while the UT techniques only detect a specific area according to designed detectors [90,91]. However, the processing technique for the picture dataset will require pixel-by-pixel processes, which typically leads to issues in processing approaches, computation efficiency, and collected dataset storage [92,93]. As for the ultrasonic phased arrays technique, the scanning areas are limited to the areas of multiple probes, though it possesses all the advantages of conventional techniques containing only one or two probes. In terms of detection depth, ultrasonic testing can detect surface and subsurface, while the infrared technique can only reflect surface profiles.

The comparisons of the different techniques demonstrate that infrared thermography can be used to attain a primary picture of the defect profiles of the inspected windows, and the detailed flaws can be evaluated by ultrasonic phased arrays. Despite the feasibility of the above techniques, these existing advanced detection systems only inspect the defects in progress, while the knowledge learned from observed corrosion or degradation phenomena is largely neglected. Making use of such knowledge can help predict aluminum window failure occurrence at an early phase and provide timely reminders for maintenance handling.

## 4. Future Outlook

The above-mentioned challenges, in terms of the unclear degradation mechanisms of aluminum and its alloys at the nanoscale as well as the lack of prediction techniques for aluminum windows, are hindering the progress of corrosion prevention and detection for aluminum windows. The utilization of molecular dynamics (MD) simulation and artificial intelligence (AI) technology shows great potential for addressing these challenges. This section highlights the importance and functions of MD simulations and AI technology for advancing aluminum window protection and inspection.

### 4.1. Using MD Simulations for Understanding Corrosion Mechanisms of Aluminum Alloy

**Figure 4:**
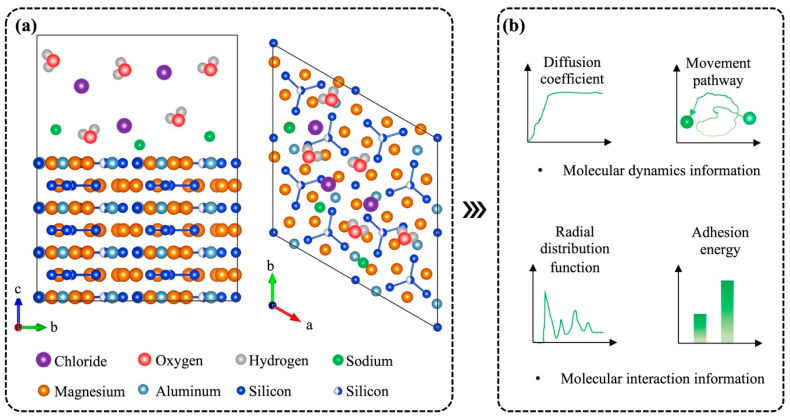
MD simulation for providing in-depth understandings of the corrosion mechanisms under different environmental factors. (**a**) The typical simulated system of aluminum alloy and environmental species. The stoichiometry is Al_3_Mg_9_Si_8_. (**b**) The atomistic information gained from MD simulations includes information of molecular dynamics and molecular interaction.

MD simulations can offer nanoscale perspectives for disclosing the interaction of aggressive species with aluminum and the movements of simulated atoms by constructing representative models with the correct forcefield [94,95,96]. This molecular-scale information helps characterize the corrosion behaviors of aluminum alloy and yields the molecular mechanisms of the corrosion [97]. Figure 4 illustrates the transformation from typical MD simulated models to useful corrosion information at the nanoscale. The successful information extraction is largely reliant upon the following two aspects, the selected model which represents the studied material systems and the forcefield which mimics atomistic interaction [98,99]. For the material systems, the aluminum materials typically used for manufacturing aluminum windows are Al-Mg-Si alloys from the 6000 series. The production of 6000 series alloy involves a general precipitation sequence. Among all the relevant products, the B’ phase is the prevalent form of metastable precipitate in over-aged Al-Mg-Si alloys [100]. Figure 4a depicts the simulated system of a B’ phase crystal structure model and aggressive species which represent different environmental factors [101]. As an example, sodium chloride solution and water molecules are embodied in the systems since they are abundant in salt and moisture environments. For the forcefield, there is not yet a suitable and verified forcefield for describing the interaction between aggressive species and aluminum alloys. It is hoped that an accurate forcefield can be developed and validated to represent the interaction based on regular processes [102,103]. After obtaining an accurate forcefield that can be applied to the studied system, information about molecular dynamics such as diffusion coefficient [104] and movement pathway [105], and molecular interaction such as radial distribution function [106] and adhesion energy [107], will become obtainable, as presented in Figure 4b. These molecular behavior indicators are then evaluated and transferred to link macroscopic information in order to interpret the corrosion behaviors of aluminum windows.

Apart from the ability to obtain comprehensive understandings of aluminum and its alloys at the nanoscale, MD simulations can aid in formulating anticorrosion strategies for aluminum products. The strategies include selecting corrosion inhibitors with high protectiveness ability and manufacturing smart anticorrosion coatings with strong interfacial adhesion for aluminum alloys. When considering the selection of inhibitors, MD simulations have the advantage over conventional experimental techniques in terms of efficiency and accuracy since the effectiveness of inhibitors stems from molecular interactions which can be obtained in a nanoscale period instead of taking months and even years by experiments [108]. In addition, MD simulations can provide guides to select the favorable inhibitors working in different environments [109]. Specifically, MD simulations can be implemented to explore the preferential molecular interactions of inhibitor-H_2_O, inhibitor-NaCl, and inhibitor-CO_2_ from molecule structure configurations. In terms of smart anticorrosion coatings, MD simulations can be utilized to investigate the interfacial performance of aluminum substrate and coating materials in different environments. Expressly, MD simulations are capable of providing molecular-level insights about the interfacial properties of organic coating and inorganic metal. These insights can help reveal the interfacial behavior between the coatings and metal surface, and help manufacture the favorable anticorrosion coatings [110,111]. Based on the excellent ability of MD simulation in terms of providing useful nanoscale information, it is believed that MD will be an efficient method to provide insight into the corrosion mechanisms and enlighten anticorrosion strategies of aluminum its alloy on a short time scale [112].

### 4.2. Using AI Technologies for Predicting the Health State of Aluminum Windows

**Figure 5:**
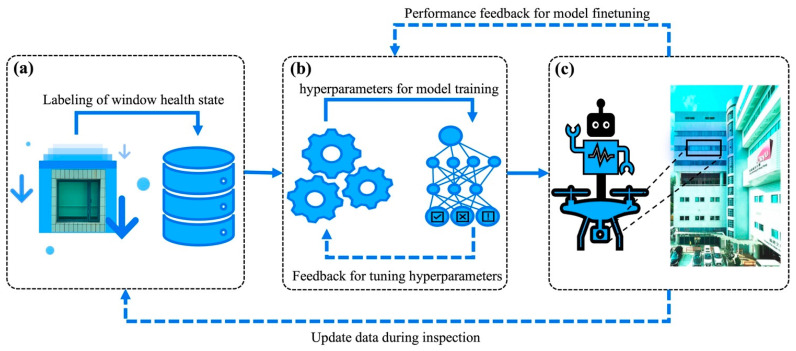
Blueprint for smart window inspection scheme using artificial intelligence technologies. (**a**) Data collection and image label. (**b**) Training model for inspecting aluminum window health state, namely recognizing broken window glass, corroded window frame, and life expectancy prediction. (**c**) Application of the trained model on drones. The images gained in the inspection process can enrich the dataset, and the model performance can provide feedback for the fine-tuning model.

AI technologies have been proven to be an effective tool for monitoring and predicting structural health conditioning in different applications, such as bridge crack monitoring and concrete properties prediction [113,114,115,116]. This effectiveness results from their ability to extract informative data that can lead to more accurate and efficient predictions [117,118,119,120]. This common advantageous feature of AI technologies is also applicable to high efficiency and accuracy in inspection and prediction tasks relating to aluminum windows. To apply AI technologies in aluminum windows, a novel and integrated system should be designed and employed. A blueprint of a typical AI-based system for aluminum window inspection is illustrated in Figure 5. The first component of the system involves collecting aluminum window image data and manually annotating the images with health state labels, as shown in Figure 5a. Although the collection and manual annotation process could be time- and labor-consuming, a large-scale dataset containing multiple aluminum window images together with their corresponding health state labels could be constructed for future usage. With the annotated data, the second component of the system involves training and tuning a learning-based model for inspection and prediction tasks, as shown in Figure 5b. A classification-based machine learning model could be built and trained to predict the health state of an aluminum window given its window image. For example, a convolutional neural network (CNN) based deep learning model can be proposed to predict the health state of the aluminum window, due to the well-proven ability of CNN and deep learning algorithms in image classification tasks [121,122]. To achieve the best model, multiple candidates for all model hyperparameters would be selected and evaluated according to the validation results during the training of the model. After the model is well-trained, the third component is aimed at utilizing it in real-world aluminum window inspection and prediction tasks, as seen in Figure 5c. Specifically, when given an image of an aluminum window collected via mobile phone apps or on-demand drones, the system would be capable of providing its current health state information and its predicted remaining service expectancy for customers’ reference.

In addition, when applying the model in real-world scenarios, more image data could be collected in order to update the dataset constructed in the first component. Specifically, since the model may not work perfectly in the beginning, manual inspection of the model prediction is still required, and the incorrectly predicted images can be added to the database again with a new annotated label assigned for further training. In addition, the feedback of the inspection and prediction results could be provided manually and periodically to the second component for model updating and finetuning [123]. For example, during the manual inspection of the model performance, feedback concerning the setting of the model hyperparameters can be used to further adjust the model structure. Then several fine-tuning steps efficiently can be conducted on the existing model without retraining the whole model from scratch [124]. It is expected that the designed system would continuously provide outstanding inspection and prediction services with high efficiency and periodically upgrade itself with up-to-date collected images and real-world performance feedback.

## 5. Conclusions

In this review, the main degradation mechanisms of aluminum windows under different aggressive environmental factors have been discussed. Among the environmental influences, chloride ions and humidity are the most detrimental factors resulting in the deterioration of aluminum windows, especially in offshore marine environments. In addition, various advanced condition monitoring techniques have been compared, with a focus on their working principles and applicable scopes. It has been discovered that infrared thermography can assist in obtaining a complete preliminary defect profile of inspected windows, while ultrasonic phased arrays technology exhibits a high level of competence in evaluating comprehensive defect information. However, the lack of insightful physical and chemical understanding of aluminum window degradation from a nanoscale perspective, and powerful inspection and prediction technologies with high efficiency and accuracy, are the key challenges that are hindering the development of window inspection. In response to these issues, future work could utilize MD simulations for a better understanding of aluminum alloy corrosion mechanisms and AI technologies to anticipate the health state of aluminum windows. When there is an accurate understanding of aluminum window degradation and the advanced technologies of aluminum window inspection, it is believed that obtaining accurate diagnoses and improving the healthcare services of aluminum windows will greatly contribute to the development of smart cities.

## Figures and Tables

**Table 1:** Advantages and limitations of existing technologies for aluminum window inspection.

Techniques	Advantages	Limitations
Ultrasonic pulse-echo (reflection) technique	Low detection cost, easy operation. Adaptability to large, irregularly shaped test specimens. Low requirements for personnel quality. Sensitive to surface and volume flaws.	Long inspection time. Single probe detection.
Ultrasonic through-transmission technique	Applicable to multi-layer materials such as those with honeycomb or foam core.	Long inspection time. Inability to attain defect location information. Requirement for parallel component surface.
Ultrasonic phased arrays technique	Reduced inspection time. Flaws’ shape and size can be directly evaluated. Increased inspection quality.	Limitations for thick components. Requires qualified personnel.
Infrared thermography technique	Fast inspection. Non-contact and non-intrusive. Portable thermal camera. No infrared radiation attenuation.	Expensive instrumentation. Requires qualified personnel. Large dataset.

## Data Availability

The raw data supporting the conclusions of this article will be made available by the authors, without undue reservation.
